# The combination of NAD^+^-dependent deacetylase gene deletion and the interruption of gluconeogenesis causes increased glucose metabolism in budding yeast

**DOI:** 10.1371/journal.pone.0194942

**Published:** 2018-03-26

**Authors:** Hiroshi Masumoto, Shigeru Matsuyama

**Affiliations:** 1 Transdisciplinary Research Integration Center, National Institute of Genetics, 1111 Yata, Mishima, Shizuoka, Japan; 2 Faculty of Life and Environmental Sciences, University of Tsukuba, 1-1-1 Tennoudai, Tsukuba, Ibaraki, Japan; Universite Paris-Sud, FRANCE

## Abstract

Metabolic engineering focuses on rewriting the metabolism of cells to enhance native products or endow cells with the ability to produce new products. This engineering has the potential for wide-range application, including the production of fuels, chemicals, foods and pharmaceuticals. Glycolysis manages the levels of various secondary metabolites by controlling the supply of glycolytic metabolites. Metabolic reprogramming of glycolysis is expected to cause an increase in the secondary metabolites of interest. In this study, we constructed a budding yeast strain harboring the combination of triple sirtuin gene deletion (*hst3*∆ *hst4*∆ *sir2*∆) and interruption of gluconeogenesis by the deletion of the *FBP1* gene encoding fructose-1,6-bisphosphatase (*fbp1*∆). *hst3*∆ *hst4*∆ *sir2*∆ *fbp1*∆ cells harbored active glycolysis with high glucose consumption and active ethanol productivity. Using capillary electrophoresis–time-of-flight mass spectrometry (CE–TOF/MS) analysis, *hst3*∆ *hst4*∆ *sir2*∆ *fbp1*∆ cells accumulated not only glycolytic metabolites but also secondary metabolites, including nucleotides that were synthesized throughout the pentose phosphate (PP) pathway, although various amino acids remained at low levels. Using the stable isotope labeling assay for metabolites, we confirmed that *hst3*∆ *hst4*∆ *sir2*∆ *fbp1*∆ cells directed the metabolic fluxes of glycolytic metabolites into the PP pathway. Thus, the deletion of three sirtuin genes (*HST3*, *HST4* and *SIR2*) and the *FBP1* gene can allow metabolic reprogramming to increase glycolytic metabolites and several secondary metabolites except for several amino acids.

## Introduction

**Fig 1:**
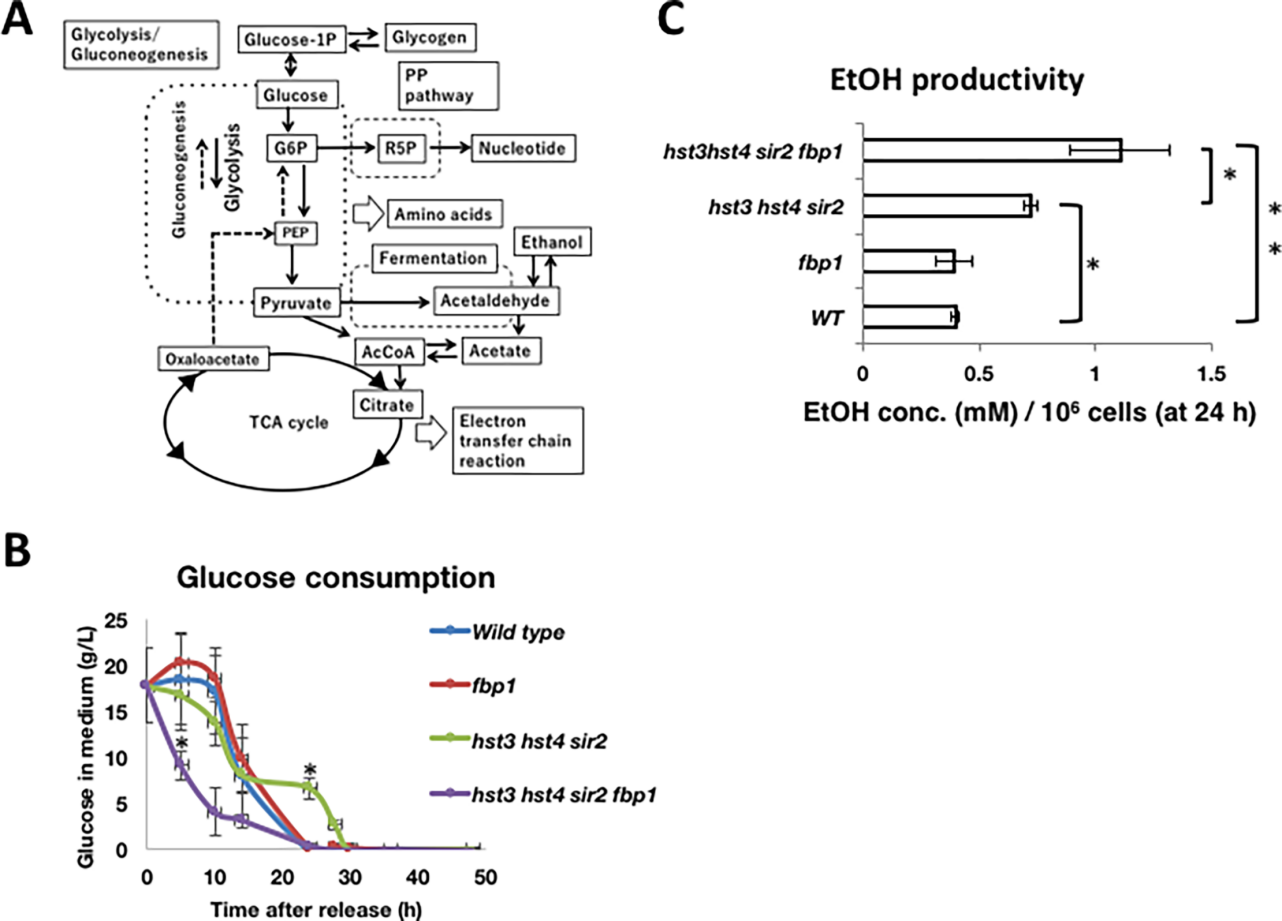
The *hst3*∆ *hst4*∆ *sir2*∆ *fbp1*∆ cells harbor active glucose metabolism but cannot contribute to cell growth. (A) Pathway of central carbon metabolism in budding yeast based on information from the *Saccharomyces* genome database website (http://www.yeastgenome.org/). G1P: glucose 1-phosphate, G6P: glucose 6-phosphate, PEP: phosphoenolpyruvate, PP pathway: pentose phosphate pathway, R5P: ribose 5-phosphate, and AcCoA: acetyl-CoA. (B) Comparison of glucose consumption among strains. Wild-type, *fbp1*∆, *hst3*∆ *hst4*∆ *sir2*∆ and *hst3*∆ *hst4*∆ *sir2*∆ *fbp1*∆ cells (1×10^6^ cells/ml) were released into fresh YPD medium and were cultured at 25°C. A small aliquot of medium was picked up following the time course to measure the cell number, glucose concentration, and ethanol concentration in medium (Panels A and B in S2 Fig). The P-value matrix contains the Mann-Whitney U-test p-value for a one-tailed test (wild-type vs. *hst3*∆ *hst4*∆ *sir2*∆ at 5 h in time course, wild-type vs. *hst3*∆ *hst4*∆ *sir2*∆ *fbp1*∆ at 24 h). P-values were calculated using ystat2008 software (Igakutosho, Japan) (*p<0.05). (C) Comparison of the ethanol productivity among yeast strains. The ethanol productivity was calculated as the concentration of ethanol released in medium per cell number at 24 h in cell cultivation (Panels A and B in S2 Fig). Multiple comparisons among strains (wild-type, *hst3*∆ *hst4*∆ *sir2*∆ and *hst3*∆ *hst4*∆ *sir2*∆ *fbp1*∆) were performed (non-repeated measures ANOVA with the Student-Newman-Keuls (SNK) test) (*p<0.05 and **p<0.01). Values are expressed as the means ± standard deviations. The experiments were repeated three times.

Metabolic engineering is the science of rewriting the metabolism of cells to enhance native products or endow cells with the ability to produce new products [1]. This engineering has the potential for wide-range application, including the production of fuels, chemicals, foods and pharmaceuticals. The combination of bioinformatics and mathematical modeling methods, which enable quantitative analysis, has facilitated the development of metabolic engineering to generate genetic modifications that alter cellular metabolism to direct the fluxes toward the product of interest [1]. Glycolysis plays a pivotal role in central carbon metabolism and may become an important target of metabolic engineering. This biochemical reaction catabolizes glucose as a carbon source and produces pyruvate, adenosine triphosphate (ATP) and various glycolytic intermediates [2]. Glycolytic metabolites are employed in secondary metabolic reactions, such as lipid and amino acid metabolism, to produce considerable species of secondary metabolites [3]. For example, glycolysis shunts into the pentose phosphate (PP) pathway, producing much-needed nucleotides for proliferation (Fig 1A). Increased glycolysis is utilized in cellular proliferation. The Warburg effect shifts from oxidative phosphorylation to aerobic glycolysis, characteristic of cancer cells [4–6]. Cancer cells drive glycolysis to generate ATP at a faster rate than oxidative phosphorylation, while producing less reactive oxygen species (ROS), nucleotides much needed throughout the PP pathway for rapid proliferation. Additionally, an increase in the fermentation of microbes contributes to human life. Budding yeast (*Saccharomyces cerevisiae*) harbors active glycolysis equipped with strong fermentation ability. This organism has been utilized to produce fermentative foods or beverages and has been recently utilized in the biofuel industry to produce biofuels such as ethanol and 1-butanol [7–9]. Metabolic engineering to activate glycolysis has the potential to achieve an increase in various secondary metabolic pathways.

Gluconeogenesis, almost the reverse biochemical reaction of glycolysis, is activated to utilize a carbon source other than glucose [3]. In budding yeast, the main gluconeogenesis-specific enzymes are fructose-1,6-bisphosphatase (Fbp1), isocitrate lyase carboxykinase (Icl1), malate dehydrogenase (Mdh2), and phosphoenolpyruvate carboxykinase (Pck1) [3, 10]. An increase in both glycolysis and glucose storage is manifested in aged yeast cells, caused by an abnormal activation of gluconeogenesis [11, 12]. Some aging-related gene deletions exhibit a metabolic status that mimics that of aged yeast cells [11, 12]. NAD^+^-dependent deacetylases, which are also called sirtuins, are involved in multiple cellular functions, including gene silencing, genome maintenance, cellular metabolism and cellular aging [13, 14]. Among the five genes in the budding yeast sirtuin family (*SIR2* and *HST1/2/3/4*), Sir2, Hst3 and Hst4 are involved in the regulation of cellular lifespan and cell metabolism [15, 16]. The increase in both glycolysis and glucose storage manifested in *hst3*∆ *hst4*∆ cells reflect enhanced gluconeogenesis [11, 12]. Additionally, *sir2*∆ cell exhibits enhanced gluconeogenesis by maintaining the acetylated form of Pck1 to prevent the conversion from phosphoenol pyruvate (PEP) to oxaloacetate [17]. Interestingly, the *TDH2* gene encodes a glyceraldehyde 3-phosphate dehydrogenase (GAPDH), and the deletion causes the interruption of gluconeogenesis [12]. Additional deletion of the *TDH2* gene coordinates the levels of glycolytic metabolites to restore the slow growth of *hst3*∆ *hst4*∆ cells [12]. This indicated that the interruption of gluconeogenesis can direct the glycolytic flux toward secondary metabolism to promote the growth of sirtuin-deleted cells.

In this study, we tried to examine whether the combination of sirtuin gene deletion and interruption of gluconeogenesis would create metabolic reprogramming of glycolysis to achieve increased secondary metabolism. We constructed a yeast strain harboring sirtuin gene deletions (*hst3*∆ *hst4*∆ *sir2*∆) combined with the interruption of gluconeogenesis (*fbp1*∆) (S1 Fig). *hst3*∆ *hst4*∆ *sir2*∆ *fbp1*∆ cells harbored active glucose metabolism with high glucose consumption and active ethanol productivity. Capillary electrophoresis–time-of-flight mass spectrometry (CE–TOF/MS) analysis revealed that not only the levels of glycolytic metabolites but also those of other secondary metabolites were dramatically increased in *hst3*∆ *hst4*∆ *sir2*∆ *fbp1*∆ cells, although several amino acids were decreased. Using the stable isotope-labeling assay for the metabolites, we confirmed that the metabolic fluxes of glycolytic metabolites were strengthened to enter the PP pathway in *hst3*∆ *hst4*∆ *sir2*∆ *fbp1*∆ cells. Thus, the deletion of both the three sirtuin genes (*HST3*, *HST4* and *SIR2*) and *FBP1* gene can allow metabolic reprogramming to increase glycolytic metabolites and several secondary metabolites except for several amino acid syntheses.

## Results

### *hst3*∆ *hst4*∆ *sir2*∆ *fbp1*∆ cells harbor active glucose metabolism

To investigate the influence of *hst3*∆ *hst4*∆ *sir2*∆ *fbp1*∆ quadruple gene deletion for glucose metabolism, we compared glucose consumption among cells. Wild-type and *fbp1*∆ cells almost simultaneously exhausted glucose in the medium until 24 h after cell culture had started (Fig 1B). *hst3*∆ *hst4*∆ *sir2*∆ cells consumed glucose more slowly than both wild-type and *fbp1*∆ cells, and much glucose remained in the medium at 24 h (Fig 1B). By contrast, *hst3*∆ *hst4*∆ *sir2*∆ *fbp1*∆ cells consumed glucose more quickly than other strains (Fig 1B). To confirm whether increased secondary metabolism occurred in *hst3*∆ *hst4*∆ *sir2*∆ *fbp1*∆ cells in connection with high glucose consumption, we examined the productivity of ethanol as a representative fermentative metabolite (Fig 1A). To evaluate the ethanol productivity of cells, we calculated the ethanol amount *per se* at 24 h in cell cultivation (Panels A and B in S2 Fig). In Fig 1C, *hst3*Δ *hst4*Δ *sir2*Δ *fbp1*Δ cells exhibited higher ethanol productivity than *hst3*Δ *hst4*Δ *sir2*Δ cells, and the ethanol productivity was almost three times that of the wild type. These data suggest that *hst3*Δ *hst4*Δ *sir2*Δ *fbp1*Δ cells directed the glycolytic flux toward secondary metabolism, including fermentation, together with high glucose consumption. Additional deletion of the *TDH2* gene coordinates the levels of glycolytic metabolites to restore the slow growth of *hst3*∆ *hst4*∆ cells [12]. Beyond that, *tdh2* gene deletion restores the growth of *hst3*∆ *hst4*∆ cells, and we expected that the *fbp1* gene deletion could restore the growth of *hst3*∆ *hst4*∆ *sir2*∆ cells. Unexpectedly, *hst3*∆ *hst4*∆ *sir2*∆ *fbp1*∆ cells arrested the growth at lower cell numbers than *hst3*∆ *hst4*∆ *sir2*∆ cells (Panel B in S2 Fig). This suggests that glycolysis and secondary metabolism do not supply metabolites sufficient to recover the growth of *hst3*Δ *hst4*Δ *sir2*Δ *fbp1*Δ cells.

### Glycolytic metabolites and nucleotides, but not amino acids, accumulate in *hst3*∆ *hst4*∆ *sir2*∆ *fbp1*∆ cells

**Fig 2:**
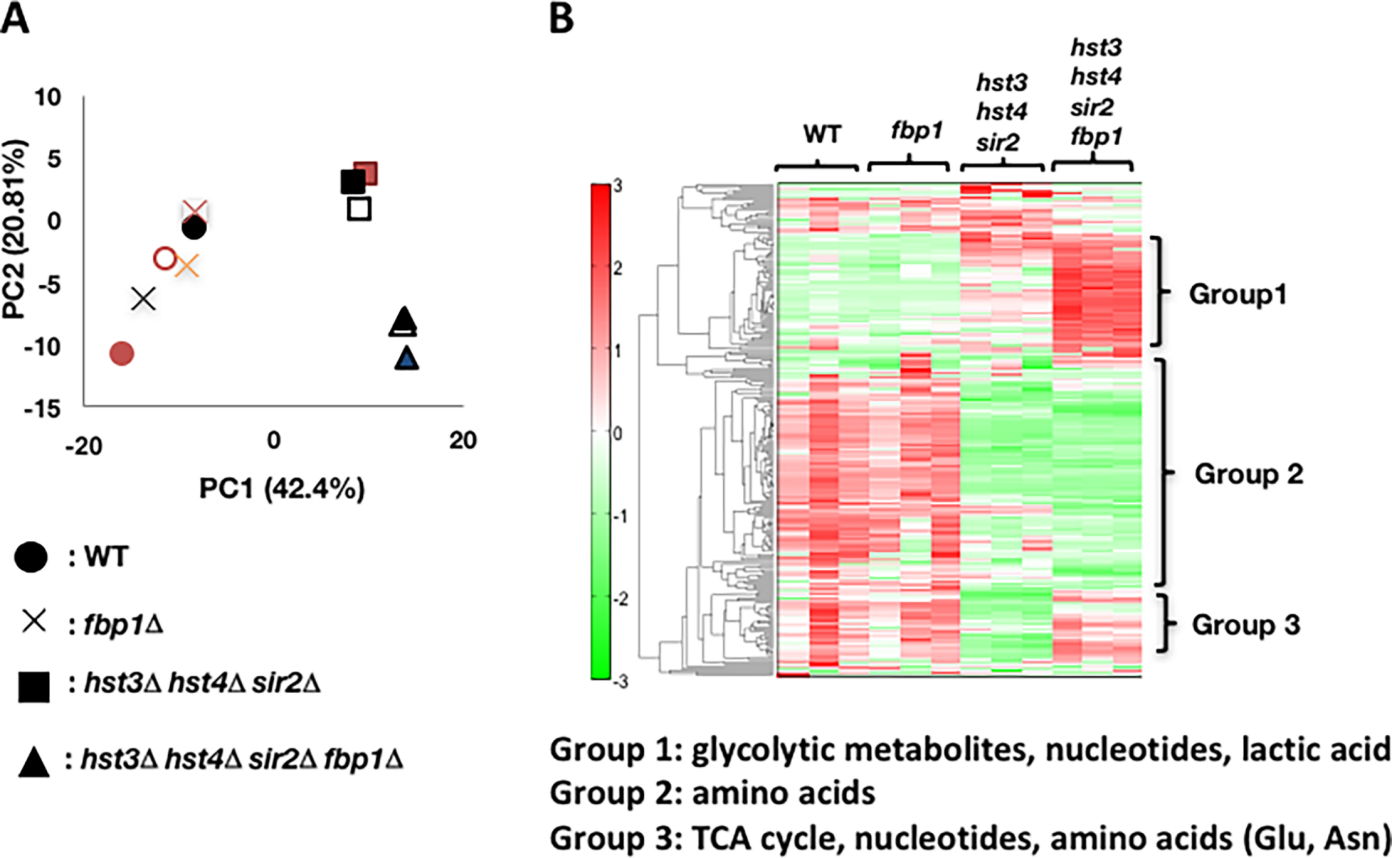
Comparison of intracellular metabolic profiles among yeast strains. (A) Principal component analysis (PCA) showing the fluctuations of the intracellular metabolites of yeast strains (wild-type, *fbp1*∆, *hst3*∆ *hst4*∆ *sir2*∆ and *hst3*∆ *hst4*∆ *sir2*∆ *fbp1*∆). PCA data (PC1 and PC2) were employed from S1 Table. (B) Heat map of hierarchical clustering of intracellular metabolite profiles from yeast strains. Red indicates a higher concentration of metabolites than the internal standard, while green indicates a lower concentration of metabolites than the internal standard. The relative amount of each metabolite per internal standard analyzed in CE–TOF/MS is listed in S1 Table. Three cell samples were employed for each yeast strain. TCA cycle: tricarboxylic acid cycle, Glu: glutamine, Asn: asparagine.

**Fig 3:**
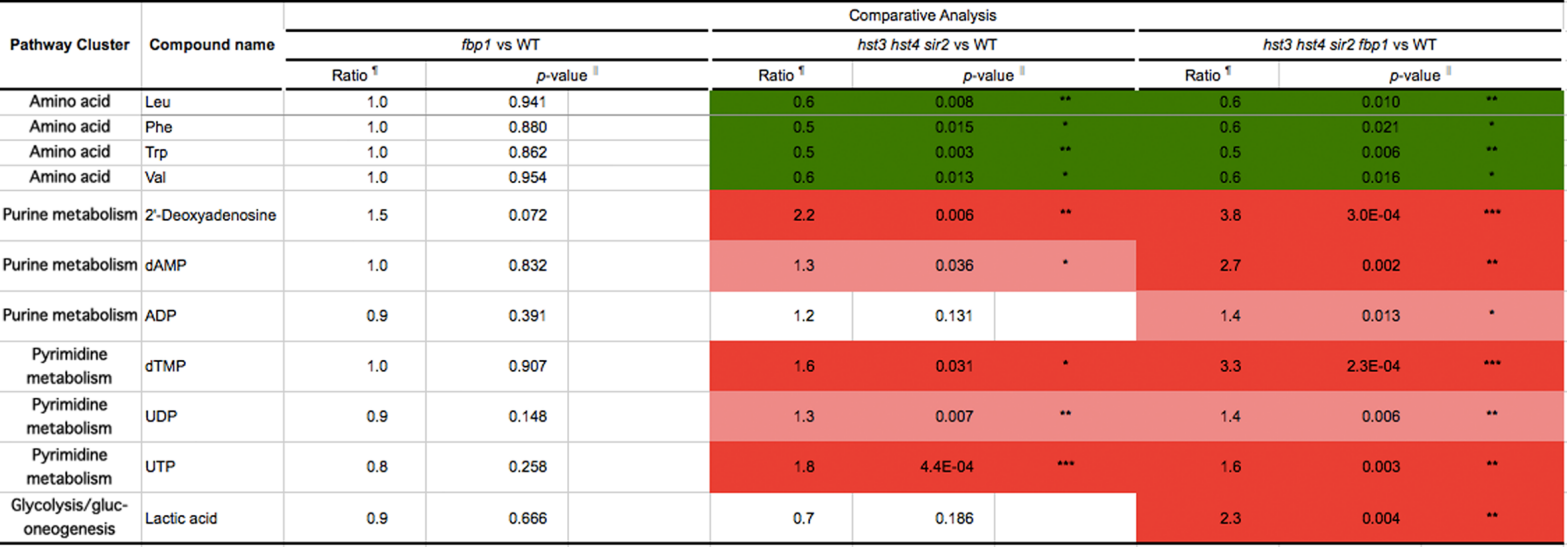
Comparative analysis of metabolites among yeast strains. The fluctuation of metabolites was calculated using wild type as the denominator. Green indicates a decrease in the metabolite levels for wild-type cells, and red indicates an increase. Representative data are employed from S3 Table. The P-value matrix contains the Welch's t-test (*<0.05, **<0.01, ***<0.001 compared with the value of wild-type cells). Leu: leucine, Phe: phenylalanine, Trp: tryptophan, Val: valine, dAMP: deoxy adenine monophosphate, ADP: adenine diphosphate, dTMP: deoxy thymidine diphosphate. UDP: uridine diphosphate, UTP: uridine triphosphate.

To investigate the cellular metabolism in *hst3*∆ *hst4*∆ *sir2*∆ *fbp1*∆ cells, we employed the CE–TOF/MS system to compare the metabolic profiles among strains (Fig 2 and S1 Table). To analyze the large dataset arising from the metabolite profiles (S1 Table), principal component analysis (PCA) was applied [18]. PC1 and PC2 explained 42.4% and 20.81% of the variance, respectively (Fig 2A and S2 Table). A bi-plot (both scores and loadings) from PCA is shown in Fig 2A. Along the first component (PC1), wild-type cells were separated from both *hst3*∆ *hst4*∆ *sir2*∆ and *hst3*∆ *hst4*∆ *sir2*∆ *fbp1*∆ cells, although they were overlapped with *fbp1*∆ cells (Fig 2A). Along the second component (PC2), *hst3*∆ *hst4*∆ *sir2*∆ and *hst3*∆ *hst4*∆ *sir2*∆ *fbp1*∆ cells were clearly separated (Fig 2A). This suggests that most of the variance in the data resulted from the combination of gene deletions. The profiles indicate that large discrepancies in metabolic activity were not only between wild-type and sirtuin gene deletion cells but also between *hst3*∆ *hst4*∆ *sir2*∆ and *hst3*∆ *hst4*∆ *sir2*∆ *fbp1*∆ cells, although there was almost similar metabolic activity between the wild-type and *fbp1*∆ strains. Next, we investigated the fluctuation of metabolites among the strains. The heat maps show the levels of metabolites and classify the metabolites as they relate to metabolism (Fig 2B and S1 Table). Both wild-type and *fbp1*∆ cells exhibited almost similar metabolic profiles: low levels of glycolytic metabolites but high levels of amino acids and relative metabolites derived from amino acid synthesis (Fig 2B and S1 Table). *hst3*∆ *hst4*∆ *sir2*∆ cells exhibited metabolic profiles different from those of both wild-type and *fbp1*∆ cells; the glycolytic metabolites and nucleotides, which are produced in purine/pyrimidine synthesis, accumulated at high levels, although several amino acids remained at low levels (Figs 2B and 3, S1 Table). *hst3*Δ *hst4*Δ *sir2*Δ *fbp1*Δ cells showed basically similar profiles as *hst3*Δ *hst4*Δ *sir2*Δ cells (Fig 2B). However, many species of metabolites, especially several species of nucleotides, were markedly increased in *hst3*Δ *hst4*Δ *sir2*Δ *fbp1*Δ cells more than in *hst3*Δ *hst4*Δ *sir2*Δ cells (Figs 2B and 3, S1 and S3 Tables). Different from *hst3*Δ *hst4*Δ *sir2*Δ cells, several metabolites in the tricarboxylic acid (TCA) cycle, amino acids (glutamine, asparagine) were accumulated in *hst3*Δ *hst4*Δ *sir2*Δ *fbp1*Δ cells (Fig 2B, S1 and S3 Tables). Additionally, lactic acid, which is synthesized from methylglyoxal, not from fermentation, in budding yeast [3], NAD^+^ and NADH, were also more abundant in *hst3*Δ *hst4*Δ *sir2*Δ *fbp1*Δ cells than in other strains (Fig 3 and S3 Table). The levels of several amino acids remained low in both *hst3*Δ *hst4*Δ *sir2*Δ *fbp1*Δ and *hst3*Δ *hst4*Δ *sir2*Δ cells (Figs 2B and 3, S3 Table). These data suggest that *hst3*Δ *hst4*Δ *sir2*Δ *fbp1*Δ cells promote many secondary metabolic pathways, including the TCA cycle, to synthesize various metabolites, except for several amino acids.

### *hst3*Δ *hst4*Δ *sir2*Δ *fbp1*Δ deletion increases the metabolic flux into the PP pathway

**Fig 4:**
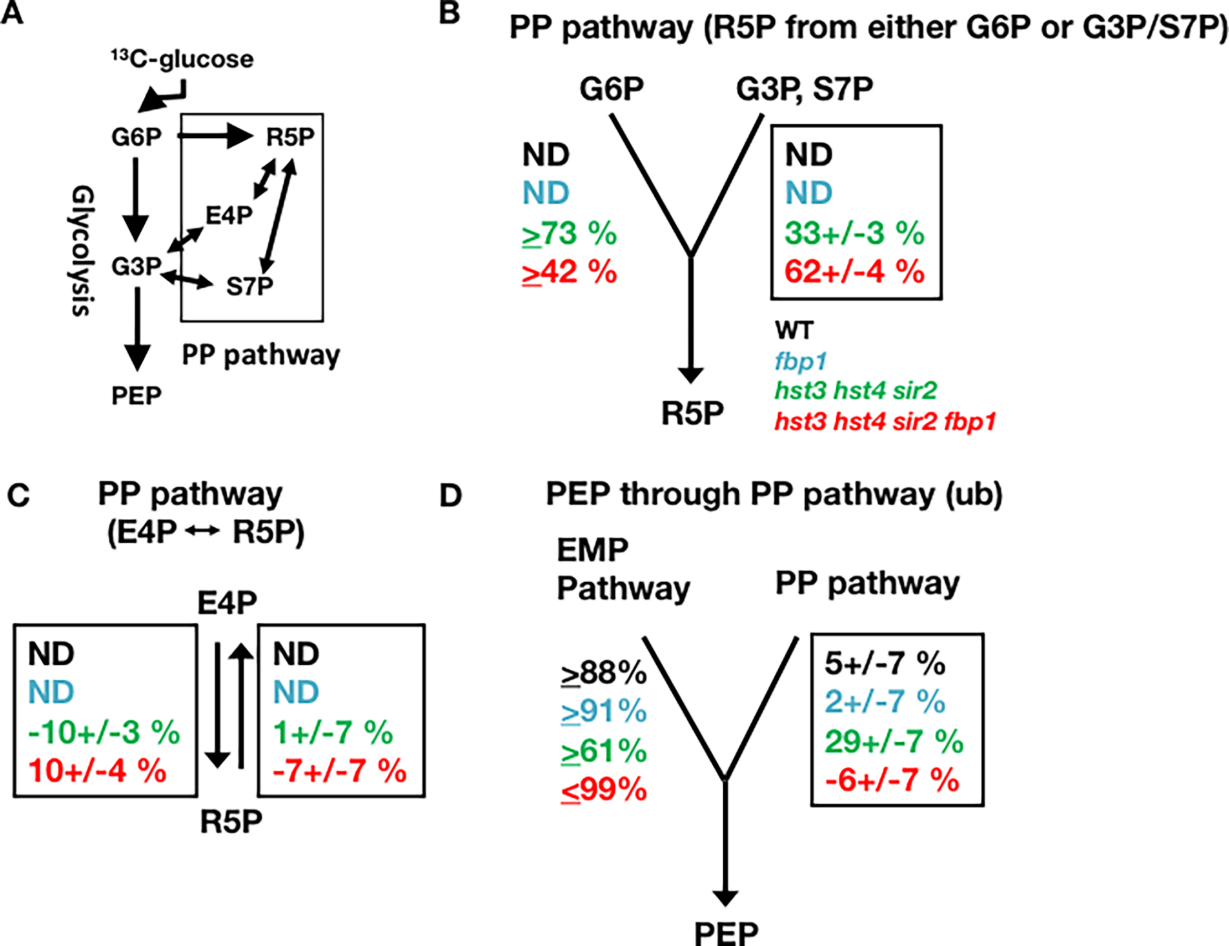
The *hst3*Δ *hst4*Δ *sir2*Δ *fbp1*Δ deletion increases the metabolic flux into the PP pathway. (A) Flux directions between glycolysis and the PP pathway. (B) Relative flux of the PP pathway (R5P from either G6P or G3P/S7P). (C) Relative flux of the PP pathway (E4P *vs*. P5P). (D) Relative flux of the glycolytic pathway (PEP through the PP pathway). Metabolic flux ratios between glycolysis and the PP pathway of wild-type (black: top values), *fbp1*Δ (blue: mid-upper values), *hst3*Δ *hst4*Δ *sir2*Δ (green: mid-lower values) and *hst3*Δ *hst4*Δ *sir2*Δ *fbp1*Δ (red: bottom values) cells during exponential growth on glucose. Relative flux in converging pathways (in %) as determined using FiatFlux for two nodes and one enzyme reaction in central carbon metabolism. The values are expressed as the means ± standard deviations. The minus values represent reverse flux in metabolic reaction. The values in the solid box were directly inferred from the analysis of local ^13^C patterns, whereas the other values are the calculated complements. The experiments were repeated twice. A representative experiment is shown. G6P: glucose 6-phosphate, G3P: glycerol 3-phosphate, PEP: phosphoenolpyruvate, R5P: ribose 5-phosphate, E4P: erythrose 4-phosphate, S7P: sedoheptulose 7-phosphate, ub: upper bounds, PP pathway: pentose phosphate pathway.

Next, we examined whether the metabolic flux was actively altered to flow into the secondary metabolism in *hst3*Δ *hst4*Δ *sir2*Δ *fbp1*Δ cells. Nucleotide synthesis needs ribose 5-phosphate (R5P), which is synthesized from G6P mainly in the PP pathway (Fig 1A). To confirm whether nucleotides were actively synthesized throughout the PP pathway or passively accumulated in *hst3*Δ *hst4*Δ *sir2*Δ *fbp1*Δ cells, we conducted metabolic flux analysis based on ^13^C-labeling experiments and subsequent gas chromatography-mass spectrometry (GC-MS) analysis [19, 20]. Because the labeling patterns of the intracellular metabolites are typically difficult to measure, reflecting their small sizes, amino acids analyses are typically employed to elucidate the labeling states in glucose metabolism [19–24]. We prepared cell extracts from cells cultivated in ^13^C-labeled glucose (Fig 4A). The cell extracts were analyzed by GC-MS, and the obtained data were further analyzed using FiatFlux software to calculate the flux ratio of several metabolic pathways located between glycolysis and the PP pathway [25, 26]. The flux ratio of any step in the PP pathway was not detected in wild-type and *fbp1*Δ cells (Fig 4B, 4C and 4D). Because the YPD medium contained many nucleotides to supply the cells, wild-type and *fbp1*Δ cells might not need to drive the PP pathway to provide R5P to purine/pyrimidine synthesis. By contrast, two major flux ratios directed toward the PP pathway from glycolysis (R5P from either G6P or G3P/S7P) were detected in *hst3*Δ *hst4*Δ *sir2*Δ cells (Fig 4B). However, the metabolites in a minor branch of the PP pathway (E4P from R5P) reversely flowed to glycolysis, indicating that metabolites were partially retained in the PP pathway in *hst3*Δ *hst4*Δ *sir2*Δ cells (Fig 4C). In addition, a portion of the metabolites in the PP pathway flowed into glycolysis in *hst3*Δ *hst4*Δ *sir2*Δ cells (Fig 4D: PEP through PP pathway). As with *hst3*Δ *hst4*Δ *sir2*Δ cells, *hst3*Δ *hst4*Δ *sir2*Δ *fbp1*Δ cells also harbored fluxes directed towards two branches of the PP pathway from glycolysis (Fig 4B: R5P from either G6P or G3P/S7P). Different from *hst3*Δ *hst4*Δ *sir2*Δ cells, however, the glycolytic metabolites flowed even into the minor branch of the PP pathway in *hst3*Δ *hst4*Δ *sir2*Δ *fbp1*Δ cells (Fig 4C: R5P from E4P). Additionally, almost no flux was directed toward glycolysis through the PP pathway in *hst3*Δ *hst4*Δ *sir2*Δ *fbp1*Δ cells (Fig 4D: PEP through the PP pathway). These data indicate that glycolytic metabolites actively flowed into the PP pathway in *hst3*Δ *hst4*Δ *sir2*Δ *fbp1*Δ cells.

## Discussion

**Fig 5:**
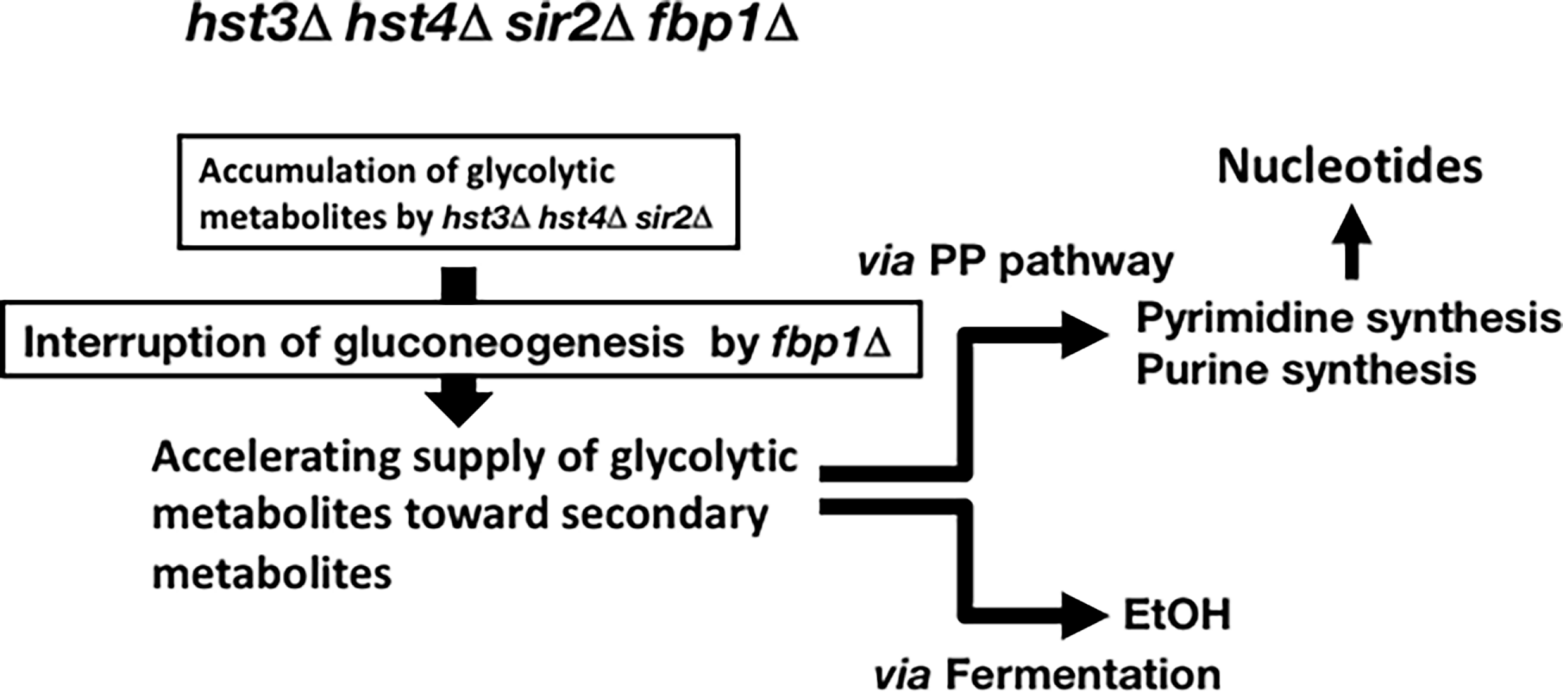
Schematic model to increase the levels of secondary metabolites in *hst3*Δ *hst4*Δ *sir2*Δ *fbp1*Δ cells.

Glycolysis manages the levels of various secondary metabolites by controlling the supply of glycolytic metabolites. Metabolic reprogramming of glycolysis, which directs the flux of glycolytic metabolites to specific secondary metabolic pathways, would be useful to increase the production of the secondary metabolites of interest. In this study, we constructed budding yeast cells that harbored the combination of a triple sirtuin gene deletion (*hst3*Δ *hst4*Δ *sir2*Δ) and the interruption of gluconeogenesis (*fbp1*∆). The *hst3*Δ *hst4*Δ *sir2*Δ *fbp1*Δ quadruple gene deletion cold also create metabolic reprogramming to direct glycolytic metabolites to achieve an increase in several secondary metabolites, except for several amino acids (Fig 5).

In wild-type cells, glycolysis is activated in the presence of glucose, in which gluconeogenesis typically remains repressed [3], and provides glycolytic metabolites for secondary metabolism. Therefore, the level of glycolytic metabolites remains low (Fig 2B and S1 Table). However, in sirtuin gene deletion (*hst3*Δ *hst4*Δ *sir2*Δ) cells, gluconeogenesis is activated even in the presence of glucose [12, 17]. This might cause conflict with active glycolysis and then accumulated glycolytic metabolites. In *hst3*Δ *hst4*Δ *sir2*Δ *fbp1*Δ cells, the interruption of gluconeogenesis by *fbp1* gene deletion may direct the metabolic flux to flow into secondary metabolism, thereby increasing the levels of secondary metabolites (Fig 5). Interestingly, several amino acids remained at low levels in sirtuin gene deletion cells (*hst3*Δ *hst4*Δ *sir2*Δ and *hst3*Δ *hst4*Δ *sir2*Δ *fbp1*Δ), although abundant glycolytic metabolites were available. This finding suggests that sirtuins (Hst3, Hst4 and Sir2) play important roles in managing a wide spectrum of amino acid syntheses. In other eukaryotes, sirtuin is involved in amino acid synthesis. In mice, Sirt4 controls leucine metabolism [27]. The target(s) of sirtuins (Hst3, Hst4 and Sir2) in several amino acid synthesis pathways may be key enzymes, leading to metabolic flux in the amino acid synthesis.

Metabolic reprogramming based on the combination of both sirtuin deletions and interruption of gluconeogenesis would be applicable to other livings and is expected to achieve a high yield of secondary metabolites, such as fermentation. Sirtuin has been widely conserved from microbes to humans and is involved in energy metabolism [14]. The enzymatic reactions and enzymes in both glycolysis and gluconeogenesis are also conserved among organisms. In budding yeast, double deletion of the *HST3 HST4* genes induces the persistent acetylation of histone H3 on lysine 56 (H3-K56) throughout the chromosome, triggering chromosomal fragility and causing harmful effects for cell viability [16, 28]. The utilization of host organisms with no histone acetylation equivalent to H3-K56 acetylation regulated by sirtuin or no obvious nucleosome structure is expected to achieve a high increase in secondary metabolism by avoiding the reduction in cell viability due to chromosomal fragility.

## Methods

### Strains and media

The parental budding yeast strain used in the present study was BY4742 [29]. The genotypes of the strains, plasmids and primers used in this study are listed in S4 Table. A yeast strain harboring a single gene deletion was commercially available from the haploid yeast open reading frame deletion collection [29] (GE Dharmacon, Lafayette, CO, USA). The scheme of yeast strain construction (HMY1370 and HMY1401) is described in S3 Fig. We employed a polymerase chain reaction (PCR)-based procedure to disrupt target genes in yeast chromosomes described previously [30, 31]. We initially constructed the HMY1369 strain by replacing the *FBP1* gene with the *natMX4* module in the *sir2*∆ strain [31]. The *nat*MX4 module was amplified using the primers HMP1016 and HMP1017 and pAG25 as a template [31]. The correct disruption of the *fbp1* gene was confirmed by PCR using both HMP923 and the *TEF1* promoter reverse primers. To construct the HMY1401 strain, the haploid strains (HMY367 and HMY1369) were crossed to generate a diploid strain (HMY367 × HMY1369) that was subsequently subjected to sporulation (S3 Fig). The *sir2* gene deletion affects the epigenetical regulation in mating loci, and the mating frequency with the *sir2*∆ strain and another MAT-type strain is quite low [32]. To isolate diploid cells after mating with the HMY367 and HMY1369 strains, we prepared the cell cultures of each HMY367 and HMY1369 strain that had been cultured in YPD liquid medium overnight at 25°C. A small portion (100 μl) of each cell culture medium was mixed in 2 ml of YPD liquid medium and was cultured again at 25°C overnight. A small portion (100 μl) of the second overnight culture was plated on YPD solid medium containing both hygromycin B and clonNAT and incubated at 25°C for 3–5 days to gain the diploid strain. The diploid strain (HMY367 × HMY1369) was streaked on YPD solid medium and was incubated at 25°C for 16 h. Cells on YPD medium were picked up and streaked on minimum SPO medium and then were incubated at 25°C for 5 days to form spores [33]. After dissection, the spores were germinated on YPD medium. Similar to the construction of the HMY1370 strain described above, the *sir2*∆ × HMY367 diploid strain was employed to sporulate and dissect to generate HMY1401 (S3 Fig). Each gene deletion was confirmed by either antibiotic resistance or histidine auxotrophy [30, 31]. Because the *hst3*∆ *hst4*∆ double deletion strains (HMY368, HMY1370 and HMY1401) harbor the PHM286 [URA3] plasmid that encodes wild-type *HST3* and prevents spontaneous DNA damage and genomic instability, we counter-selected cells for loss of the PHM286 plasmid by the addition of 5-fluoroorotic acid (5-FOA) prior to use in subsequent experiments.

We routinely employed YPD liquid medium containing 2% Bacto peptone (BD Difco, Franklin Lakes, NJ, USA), 1% yeast extract, and 2% glucose. YPD solid medium contained agarose at a final concentration of 2%. The composition of SD medium and minimum SPO medium has been previously described [33]. We employed YPD media supplemented with the following antibiotics: G418 (Sigma-Aldrich, St. Louis, MO, USA) at a final concentration of 100 μg/ml for the *kan* gene, 200 μg/ml of hygromycin B (Wako, Osaka, Japan) for the *hph* gene and 100 μg/ml of ClonNAT (Werner Bioagents, Germany) for the *nat*MX4 gene. SD-histidine medium was employed to select the *his5*^+^ strain [30, 31].

### Growth rate, ethanol and glucose quantification assay

The cells were cultured overnight in YPD medium at 25°C and were subsequently suspended in YPD medium (1×10^6^ cells/ml) and cultured at 25°C with shaking. An aliquot (1 ml) of the culture was sampled following the time course. The culture was centrifuged to collect the medium. The medium was transferred to a new Eppendorf tube and was boiled for 3 min to inactivate the enzymes that consume ethanol and glucose in the medium. The boiled culture medium was subsequently stored at -80°C until further use. Simultaneously, the cell number was determined every 24 h using a Z-1 Coulter Counter (Beckman-Coulter, Brea, CA, USA). The boiled medium (10 μl) was assayed using the Ethanol Colorimetric/Fluorometric Assay Kit or Glucose Colorimetric/Fluorometric Assay Kit (BioVision, Milpitas, CA, USA) according to the manufacturer’s instructions. Fluorescence changes (Ex/Em = 535/587 nm) were monitored using an Infinite F200 fluorescence microplate reader (TECAN, Männedorf, Switzerland). At least three replicates were analyzed for each strain.

### Metabolome analysis

#### Cell preparation for metabolome analysis

The cells were cultured overnight in YPD medium at 25°C, subsequently suspended (5×10^6^ cells/ml) in SD medium (2% glucose) and cultured at 30°C with continuous shaking for 3 h. Because *hst3 hst4* double gene deletion causes temperature sensitivity[15], HMY1370 (*hst3*∆ *hst4*∆ *sir2*∆ *fbp1*∆) and HMY1401 (*hst3*∆ *hst4*∆ *sir2*∆) cells were cultured at 25°C. The cells (OD_600_ = 20) were routinely employed for CE–TOF/MS analysis. Metabolite extraction, measurement of ionic metabolites by CE–TOF/MS, quantification of metabolites and data analyses were performed as previously described [12]. Principal component analysis (PCA) was calculated by Samplestat Ver. 3.1.4 (HMT, Tsuruoka, Japan). A heat map of the hierarchical clustering of intracellular metabolite profiles from yeast strains was constructed by PeakScat ver. 3.18 (HMT, Tsuruoka, Japan) [34–36].

### Metabolic flux ratio analysis by GC–MS

#### Cell culture and preparation of cell samples

The cells were inoculated into 5 ml of SD medium and were cultured at 25°C overnight. To enter the log-phase, an aliquot of precultured cells was resuspended in 10 ml of SD medium and were cultured at 25°C for 3 h. To incorporate ^13^C into cellular metabolites, 0.4% D-glucose-^13^C_6_ (Sigma-Aldrich, St. Louis, MO, USA) and 1.6% of natural D-glucose were used as carbon sources in the SD medium. The cells were harvested and suspended in 10 ml of SD medium containing ^13^C-glucose and were subsequently cultured at 25°C until an OD_600_ of 0.5 was reached.

#### Sample preparation for GC–MS analysis

The samples were prepared as previously described [25] with minor modifications. Briefly, the cells were collected and washed twice with distilled water. The cell pellets were suspended in 200 μl of 6 M HCl and were heated at 105°C overnight. The cell samples were dried at 95°C in the hood and stored at -20°C until further use. Dried hydrolysates were transferred into conical vials using distilled water. The vials were placed in a glass tube oven (GTO-250RN, SIBATA, Saitama, Japan) and dried at 50°C for 1 h under an aspirator vacuum over P_2_O_5_. The sample was mixed with 40 μl of *N*-*tert*-butyldimethylsilyl-*N*-methyltrifluoroacetamide (Sigma-Aldrich, St. Louis, MO, USA) and 40 μl of dimethylformamide (DMF) (Wako, Osaka, Japan). The vials were tightly capped and incubated at 100°C for 1 h. The sample was analyzed by GC–MS (GC unit: Agilent Technologies 6890N; MS unit: JEOL MS-600H) equipped with a DB-5MS column (0.25 mm × 25 m, 0.25-μm film thickness; Agilent Technologies, Santa Clara, CA, USA). The oven temperature was initially programmed at 160°C for 1 min and was subsequently increased to 320°C at a rate of 20°C/min (held for 5 min). The MS data were obtained over a scan range of 180–550 amu for 0.29 sec. The GC–MS data were analyzed using FiatFlux software to calculate the flux ratio[25, 26].

## Supporting information

S1 Fig Fbp1 is involved in gluconeogenesis. Fbp1 is involved in the biochemical reaction from F1,6P_2_ to F6P in gluconeogenesis. P: Phosphate, P_2_: diphosphate.
(TIFF)

S2 Fig Ethanol concentration in medium among strains. (A) Cells (1×10^6^ cells/ml) were released into fresh YPD medium, and a small aliquot of YPD medium was sampled to measure the ethanol concentration in the medium 24 h after cells were released. (B) Growth curve of yeast cells. The experiments were repeated three times. All graphs represent the means of triplicate results. The values are expressed as the means ± standard deviation.
(TIFF)

S3 Fig Scheme of strain construction (HMY1370 and HMY1401). (TIFF)

S1 Table Concentration of each metabolite analyzed by CE–TOF/MS. The fluctuation of metabolites was calculated based on the levels of internal standard substances. Green indicates a lower level than standard, and red indicates a higher level.
(XLSX)

S2 Table Principal component analysis (PCA) using the data of CE–TOF/MS. Green indicates the decrease in metabolites for internal control, and red indicates the increase.
(XLSX)

S3 Table Comparative analysis of metabolites among yeast strains. The fluctuation of metabolites was calculated by wild type as the denominator. The green color indicates the decrease in metabolites for wild-type cells, and red indicates the increase. The P-value matrix contains the Welch's t-test (*<0.05, **<0.01, ***<0.001) compared with the value of wild-type cells).
(XLSX)

S4 Table Yeast strains, plasmids and primers used in this study. (XLSX)
