# Supplementary figures and images for: The combination of NAD+-dependent deacetylase gene deletion and the interruption of gluconeogenesis causes increased glucose metabolism in budding yeast
Source: PLoS One. 2018 Mar 26;13(3):e0194942. doi: 10.1371/journal.pone.0194942 (PMC5868833; doi:10.1371/journal.pone.0194942)

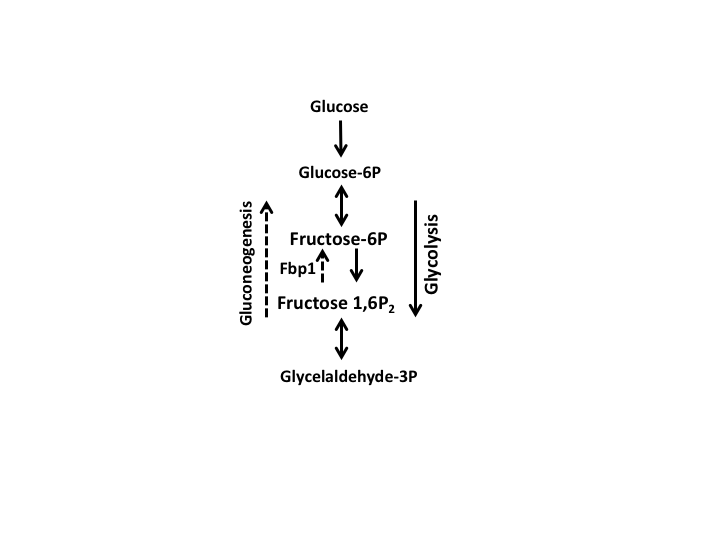

Supplement: S1 Fig — Fbp1 is involved in the biochemical reaction from F1,6P2 to F6P in gluconeogenesis. P: Phosphate, P2: diphosphate. (TIFF) [file pone.0194942.s001.tiff]

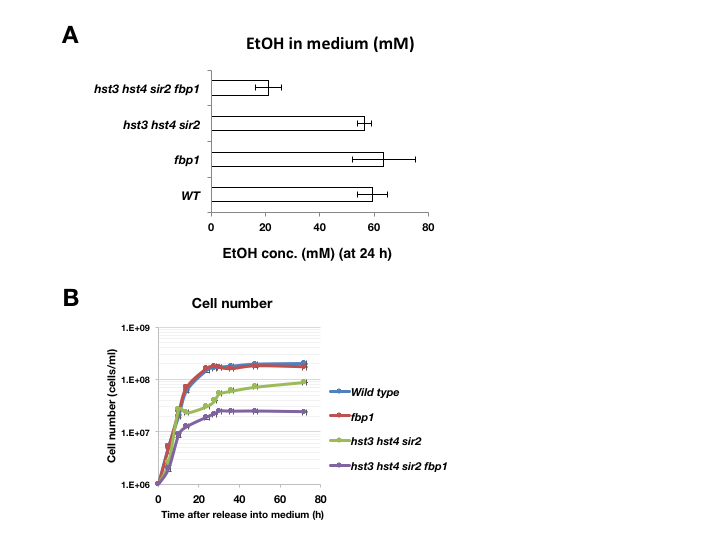

Supplement: S2 Fig — (A) Cells (1×10^6^ cells/ml) were released into fresh YPD medium, and a small aliquot of YPD medium was sampled to measure the ethanol concentration in the medium 24 h after cells were released. (B) Growth curve of yeast cells. The experiments were repeated three times. All graphs represent the means of triplicate results. The values are expressed as the means ± standard deviation. (TIFF) [file pone.0194942.s002.tiff]

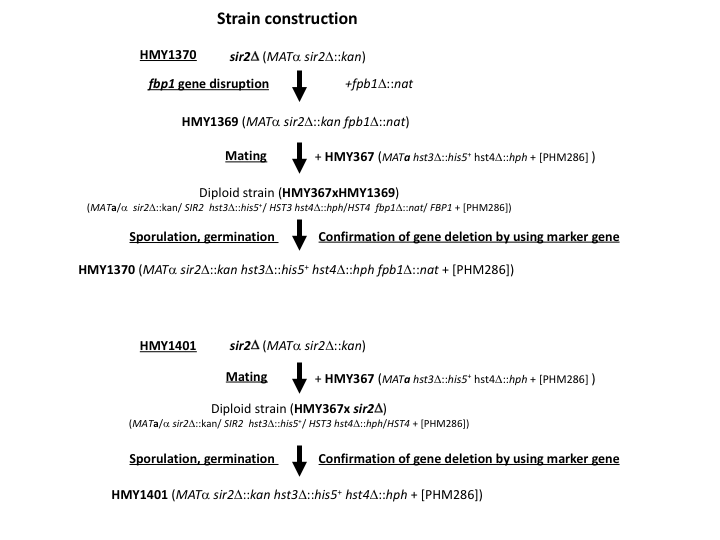

Supplement: S3 Fig — (TIFF) [file pone.0194942.s003.tiff]
